# Effects of Steroids on Quality of Recovery and Adverse Events after General Anesthesia: Meta-Analysis and Trial Sequential Analysis of Randomized Clinical Trials

**DOI:** 10.1371/journal.pone.0162961

**Published:** 2016-09-15

**Authors:** Takahiro Mihara, Tomoko Ishii, Koui Ka, Takahisa Goto

**Affiliations:** 1 Department of Anesthesiology, Kanagawa Children’s Medical Center, Mutsukawa 2-138-4, Minami-ku, Yokohama, Japan; 2 Department of Anesthesiology and Critical Care Medicine, Yokohama City University Graduate School of Medicine, 3–9 Fukuura, Kanazawa-ku, Yokohama, Japan; Copenhagen University Hospital, DENMARK

## Abstract

**Background:**

Quality of recovery (QoR) after surgery is a relevant outcome. The early postoperative quality of recovery of a patient can be determined using the QoR-40 questionnaire. The aim of this meta-analysis and Trial Sequential Analysis was to determine if perioperative administration of glucocorticosteroids improved patients’ quality of recovery after general anesthesia and if adverse events occurred.

**Methods:**

We searched six databases, including trial registration sites. Randomized clinical trials reporting the efficacy of glucocorticosteroids on quality of recovery evaluated using the QoR-40 after general anesthesia were eligible. The QoR-40 data were combined as the mean difference with confidence intervals using a random-effects model. The I^2^ statistic was used to assess heterogeneity. The quality of the trials was evaluated using the Cochrane methodology. Moreover, Trial Sequential Analysis was carried out to prevent the inflation of type 1 errors caused by multiple testing and sparse data. We also assessed adverse events.

**Results:**

Three randomized clinical trials (totaling 301 patients) were analyzed. The results from one published and four unpublished randomized clinical trials were unavailable. Dexamethasone was investigated in all three trials, and the results suggested that it significantly improved QoR-40 at postoperative day one scores compared with placebo (mean difference [95% confidence interval]: 14.2 points [10.4 to 18.1]; P < 0.001; I^2^ = 0%). We could not conduct sensitivity analysis because of the absence of trials with low risk of bias. The Trial Sequential Analysis-adjusted confidence interval was -1.6 to 30.0, indicating that further trials are required. The reporting of adverse events was insufficient.

**Conclusions:**

These findings indicate that perioperative dexamethasone administration may improve short-term (i.e., one day) quality of recovery after general anesthesia and surgery. We need more randomized clinical trials with low risk of bias assessing the effects of glucocorticosteroids on quality of life, other outcomes, and adverse events. Updated systematic reviews should then be conducted.

**Trial Registration:**

University Hospital Medical Information Network Clinical Trials Registry: UMIN000015678.

## Introduction

Quality of recovery (QoR) in the postoperative setting has become an important outcome measure. The QoR-40 [1] is a global measure of postoperative recovery, and is recommended for assessing postoperative QoR [2,3]. The QoR-40 questionnaire was reported to cover all eight criteria that are required for QoR measurement [2]. A previous meta-analysis evaluated and confirmed the validity of the QoR-40 for assessing QoR following various types of surgeries [4].

The use of glucocorticosteroids is recommended to prevent postoperative nausea and vomiting in a recently published guideline [5]. Furthermore, the glucocorticosteroids-related attenuation of postoperative pain, as well as improvements in mood and fatigue have been demonstrated [6–9]. Naturally, these positive effects of glucocorticosteroids are considered to improve QoR, as physical comfort, pain, and the emotional state are dimensions assessed by the QoR-40. The QoR-40—a patient-centered measurement tool including every aspect required for QoR measurement—would be a more important outcome measurement tool than reporting only one aspect of physiological outcomes such as postoperative nausea and vomiting or pain. In other words, perioperative use of steroids would not be recommended if it decreased the QoR-40 even if it improved pain or PONV. Therefore, we aimed to conduct a meta-analysis on this topic.

Although the risk of false-positive results from meta-analyses has been estimated to be as high as 35–50% [10,11], it can be reduced by using a Trial Sequential Analysis (TSA) [12]. Therefore, we consider TSA an essential tool for meta-analysis [13], and we used TSA to minimize the high risk of false-positive results.

We carried out a meta-analysis and TSA to estimate the effect of steroids on QoR and adverse events after surgery.

## Materials and Methods

We referred to the Cochrane Handbook [14] and recommendations from the PRISMA statement [15,16]. Our study protocol and analysis methods were pre-specified and registered in the University Hospital Medical Information Network Clinical Trials Registry (UMIN000015678).

### Database Search

The following four databases and two pre-registration sites were searched: Embase, MEDLINE, the Web of Science, the Cochrane Database, clinicaltrials.gov, and the UMIN Clinical Trials Registry (last searched on Jun 15, 2016). Moreover, we searched the reference lists of the retrieved full articles. The PubMed search strategy is provided in the supporting information (S1 Supporting Information).

Two reviewers (TI and TM) independently made evaluations on the title and abstract of all studies initially identified as candidate articles for inclusion in the current study. The full texts of studies that were considered eligible by at least one reviewer were further evaluated. Finally, studies that satisfied the criteria were assessed by two reviewers (TI and TM).

### Study eligibility

All randomized clinical trials comparing glucocorticosteroids such as dexamethasone, methylprednisolone, and hydrocortisone with a placebo that evaluated QoR after general anesthesia using QoR-40 scores were included. We excluded animal investigations, case reports, and reviews. We did not restrict eligibility by language, method of anesthesia, or type of surgery.

Our primary outcome was the effect of steroids compared with that of a placebo on postoperative QoR improvements. The secondary outcome was the occurrence of glucocorticosteroid-related adverse effects (e.g., infection and hyperglycemia).

### Data abstraction

The following data were abstracted: (1) weight; (2) age; (3) American Society of Anesthesiologists physical status; (4) method of anesthesia; (5) surgery type; (6) type of steroids; (7) drug administration route; (8) dose of steroids; (9) timing of the steroid administration; (10) control drug (e.g., no treatment or placebo); (11) number of participants; (12) QoR measurement method; (13) QoR results in the steroid and control groups; and (14) any adverse effects.

We aimed to use postoperative day 1 QoR-40 scores where available. We used the next closest time point if day 1 QoR-40 scores were not available. In addition, we aimed to extract scores from postoperative days 3–5 QoR-40. We recorded the mean and standard deviation (SD) of continuous data. When data were presented as medians and ranges, the mean and SD were calculated as per Hozo et al. [17]. All steroid groups were combined into a single group for studies that included multiple steroid groups with different doses, as recommended by the Cochrane Handbook [14]. Two reviewers (TI and TM) independently abstracted and cross-checked the data.

### Assessment of risk of bias

We evaluated the trial risk of bias with the Cochrane tools [14]. We evaluated the following domains: method of “random sequence generation,” “allocation sequence concealment,” “blinding,” “incomplete outcome,” “selective reporting,” and “other biases.” The “risk of bias” was classified into three categories: “low,” “high,” or “unclear.” Two authors (TI and TM) evaluated the risks of bias in the trials. Trials with one or more risk of bias domain that was unclear or at high risk of bias were considered as trials at high risk of bias [18–21].

### Assessment of the quality of evidence

We used “the Grading of Recommendations Assessment, Development, and Evaluation (GRADE)” approach [22] to grade the “quality of evidence” of the primary outcome. In the GRADE approach, the following domains are evaluated: “inconsistency,” “the risk of bias,” “indirectness,” “publication bias,” and “imprecision of the results.” The GRADE approach classifies the quality of evidence into four categories: “high,” “moderate,” “low,” or “very low.” We have formulated a summary of findings table using GRADEpro software (version 3.6 for Windows; available from http://ims.cochrane.org/revman/gradepro).

### Statistical analyses

The QoR-40 score data were summarized. The mean difference (MD) and confidence interval (CI) were used. We assessed heterogeneity between trials using the I^2^ statistic. A random-effects model (DerSimonian and Laird methods [23]) was used to pool the effect of steroids reported in each randomized clinical trials. We used forest plots to visualize the results of each randomized clinical trials and the meta-analyzed results. Small study effects were planned evaluated using a funnel plot when the number of studies was greater than nine. The asymmetry test (Begg’s test [24]) was planned applied for the funnel plot. We planned to carry out an additional sensitivity analysis limiting the data to trials with low risk of bias.

Additionally, we conducted TSA [12,25–29]. TSA can reduce false-positive results caused by multiple testing and sparse data. We calculated the required information size (RIS), the trial sequential monitoring boundaries for benefits and harms, and the TSA-adjusted CIs. The risk of type 1 errors was set at 0.05 with a power of 0.9. The variance was calculated from the data obtained from the included trials. A clinically meaningful anticipated MD of the QoR-40 score was set at 5 points (3% of the QoR-40 scale range of 160) and 15 points (9% of the QoR-40) as a sensitivity analysis. Although the heterogeneity in our results was 0%, we applied the anticipated heterogeneity at 50% [30]. This is because the effect of dexamethasone observed in the trial [31], which we could not include in the meta-analysis, seemed to be lower than that in other trials included in the meta-analysis. If the 95% CI included a value of 0, we considered the difference statistically non-significant. We used TSA Viewer version 0.9 β [28] (available at www.ctu.dk/tsa) to conduct the TSAs. R software version 3.0.2. (The R Foundation for Statistical Computing; Vienna, Austria) was used for other statistical analyses.

## Results

### Search results

**Fig 1:**
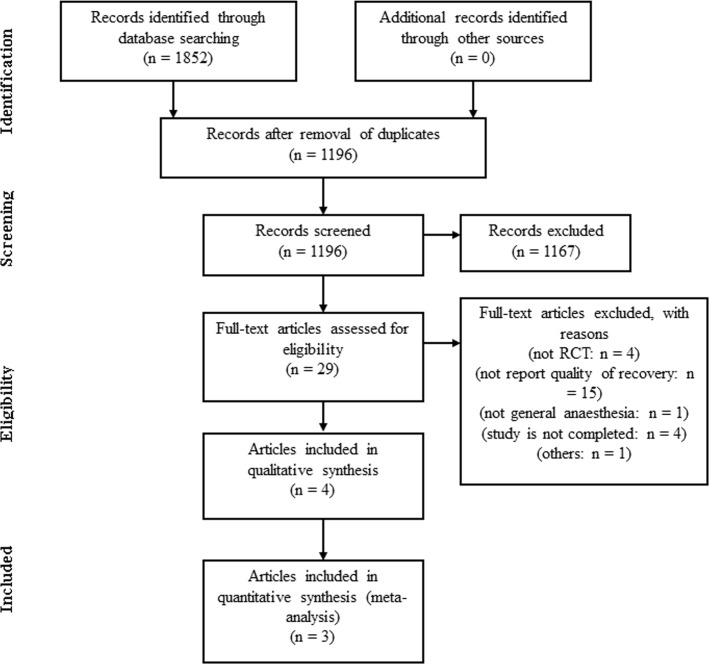
Flow diagram of the systematic review process.

The search of the electronic databases identified 1196 citations. After crosschecking titles and abstracts, the full texts of 29 articles were examined in detail. Four randomized clinical trials [31–34] that included QoR-40 scores were eligible. However, we could only analyze three randomized clinical trials [32–34] (totaling 301 patients) because one study [31] did not report the absolute value of QoR-40 and we did not try to obtain their data. We were unable to include the other four randomized clinical trials [35–38] that were initially selected from the clinical trial registration sites because they were in progress and the results were not available at the time of our meta-analysis (Fig 1). The PRISMA checklist is provided in S1 Table.

### Study characteristics

The features of the randomized clinical trials included in this meta-analysis are shown in Table 1. In all studies, intravenous (i.v.) dexamethasone was administered before initiation of the surgical incision. Dexamethasone dosages ranged from 0.05 mg · kg^-1^ to 0.1 mg · kg^-1^. The QoR-40 was scored on postoperative day 1 in all four studies. There was no study evaluating the QoR-40 score on days 3–5 after surgery.

**Table 1:** Summary of the randomized clinical trials.

Source	ASA-PS	Mean age (SD)	Total No. patients	Surgery	Type of Anesthesia Maintenance	Post-operative Analgesia	Timing of Scoring	Study Drug	Dose of Study Drug	Timing of Study Drug	Postoperative Infection	Postoperative Hyperglycemia	Other outcomes
De Oliveira, 2011 [27]	1–2	37 years (9.9)	106	Outpatient gynecological laparoscopy	Sevoflurane	Hydromorphone, ketorolac (in the hospital), ibuprofen, or hydrocodone plus acetaminophen (after discharge)	Day 1	Dexamethasone	0.1 or 0.05 mg kg^-1^	In the preoperative holding area	Not reported	Not reported	Sore throat, coughing, and hoarseness was reduced in the dexamethasone 0.1 mg kg-1 group.
Murphy, 2011a [29]	1–3	50.3 years (16.1)	91	Outpatient laparoscopic cholecystectomy	Sevoflurane	Hydromorphone (PACU)/oral acetaminophen + hydrocodone (ASU) as a rescue drug	Day 1	Dexamethasone	8 mg	Approximately 60 minutes before the anticipated time of the surgical incision	No patients required readmission for complications related to surgery, such as wound infection	Not reported	Increased appetite was observed 6/46 in the dexamethasone and 0/45 in control group. The incidence of sleeplessness, headache, stomach pain, and negative mood change were not different.
Murphy, 2011b [28]	2–4	63.1 years (12.7)	104	CABG with CPB or single valvar repair/replacement	Isoflurane + propofol (from sternal closure)	Intravenous boluses of morphine	Day 1 & Day 2	Dexamethasone	8 mg	Approximately 45 minutes before surgical incision and at the initiation of CPB	Infection was observed 1/49 in control and 0/60 in dexamethasone group	Serum glucose concentrations was higher in the dexamethasone group, but it was not statistically significant.	Postoperative cardiac arrhythmias were observed 5/58 in dexamethasone and 5/49 in control group. Postoperative shivering was observed 0/58 in dexamethasone and 8/49 in control group.
Pauls, 2015 [30]	1–3	62.5 years (9.0)	63	major vaginal reconstructive surgery	Not reported	Hydromorphone, ketorolac	Day1	Dexamethasone	8 mg	60 minutes prior to surgery	No subjects had fever, ileus, or infections in the perioperative period	Not record blood glucose levels on all patients	Anemia was observed 1/27 in dexamethasone and 0/36 in control group.

ASU, avocado/soybean unsaponifiables; ASA-PS, American Society of Anesthesiologists physical status; CPB, cardiopulmonary bypass; CABG, coronary artery bypass graft; PACU, post-anesthesia care unit; SD, standard deviation

### Intervention effects

**Fig 2:**
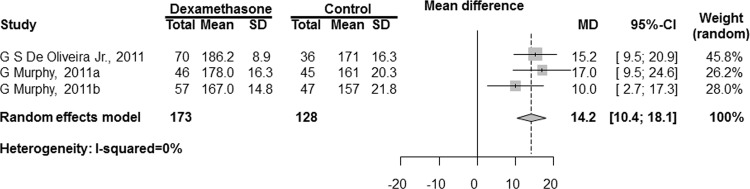
Meta-analysis of the mean difference in QoR-40 scores between the dexamethasone and control groups. SD, standard deviation; MD, mean difference; CI, confidence interval.

The combined results are shown in Fig 2. The QoR-40 scores were significantly increased with dexamethasone administration compared with placebo using traditional naive 95% CIs (MD [95% CI]: 14.2 points [10.4 to 18.1]; P < 0.001; I^2^ = 0%). We could not conduct a sensitivity analysis because there was no trial at low risk of bias.

**Fig 3:**
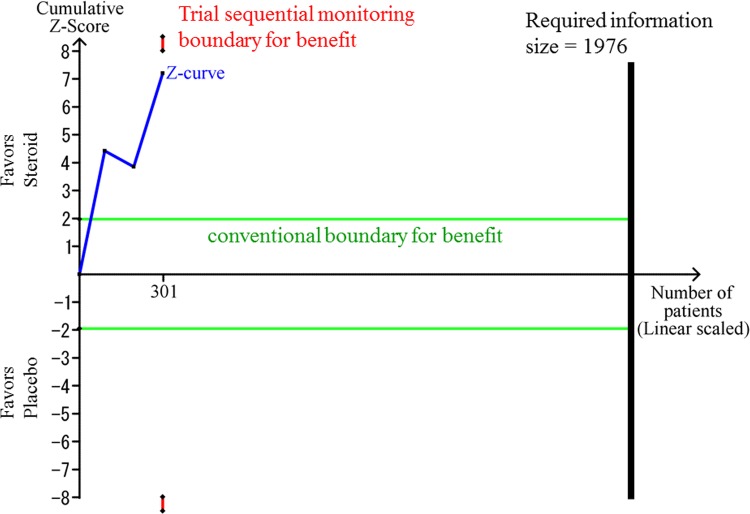
The Trial Sequential Analysis for the effect of glucocorticosteroids compared with placebo. The risk of type 1 errors was set at 0.05 with a power of 0.9 when the Trial Sequential Analysis was performed. The variance was calculated from the data obtained from the included trials. A clinically meaningful anticipated mean difference of the QoR-40 score was set at 5 points. We applied the anticipated heterogeneity at 50%.

The TSA-adjusted CI was -1.6 to 30.0 points, showing that the effect of steroids on QoR was not statistically significant. The cumulative Z-score did not cross the trial sequential monitoring boundary for benefit (Fig 3). The accrued information size (n = 301) reached only 15.2% of the estimated RIS (n = 1976).

**Fig 4:**
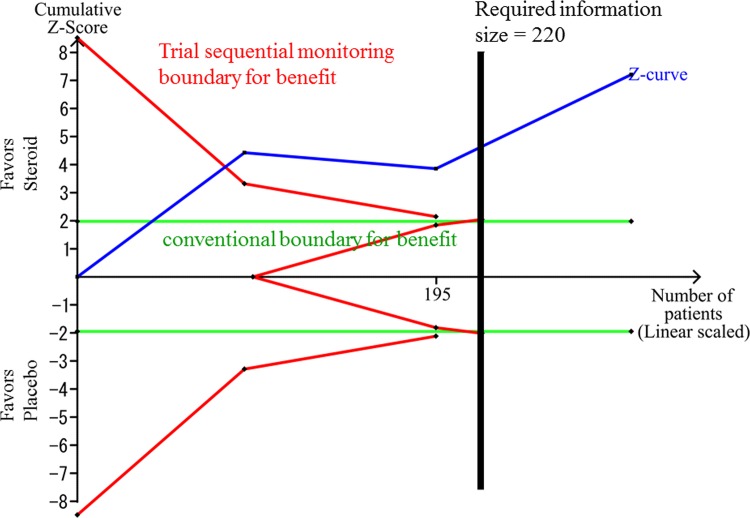
The sensitivity analysis of the Trial Sequential Analysis for the effect of glucocorticosteroids compared with placebo. The risk of type 1 errors was set at 0.05 with a power of 0.9 when the Trial Sequential Analysis was performed. The variance was calculated from the data obtained from the included trials. A clinically meaningful anticipated mean difference of the QoR-40 score was set at 15 points. We applied the anticipated heterogeneity at 50%.

The sensitivity analysis, setting a minimal relevant difference of QoR-40 score at 15 points, indicated that TSA-adjusted CI was 10.4 to 18.1 points and the cumulative Z-score crossed the trial sequential monitoring boundary for benefit (Fig 4). The accrued information size reached 137% of the estimated RIS (n = 220).

A study [31] reported the relative value of the QoR-40 score by dividing the postoperative score with the preoperative score, but did not report the absolute values; hence, it was excluded from the meta-analysis. In this study, a non-significant effect of dexamethasone on improving the QoR-40 was reported. The emotional state domain was significantly improved whereas the pain domain was not. We speculate that there may be some error in the data collection process in this trial because the QoR-40 score in pain domain was reported to be improved by 42.5% and 25.6% after surgery in control and dexamethasone groups, respectively.

Postoperative infection was not observed in the dexamethasone group in two trials [31, 33], whereas it was not reported in the remaining two trials [32, 34] (Table 1). One trial reported that increased appetite was significantly increased in the dexamethasone group [34]. TSA for these adverse events was not conducted due to the paucity of data.

### The risks of bias of the included trials

The risks of bias in the included trials are summarized in Table 2. All trials were considered at high risk of bias. The reasons for the bias assessment are provided in S2 Table.

**Table 2:** The risks of bias of the included trials.

Source	Sequence generation	Allocation concealment	Patients blinded	Health care providers blinded	Data collectors blinded	Outcome assessors blinded	Incomplete outcome data	Selective reporting	Other bias	Overall risk
De Oliveira, 2011 [32]	Low	Low	Low	Low	Low	Low	Low	Unclear	Low	High
Murphy, 2011a [34]	Low	Low	Low	Low	Low	Low	Unclear	Unclear	Low	High
Murphy, 2011b [33]	Low	Low	Low	Low	Low	Low	Unclear	Low	Unclear	High
Pauls, 2015 [31]	Low	Low	Low	Low	Low	Low	High	Unclear	High	High

### Quality of the evidence

The effect of glucocorticosteroids on QoR-40 compared with placebo was graded as “very low” (Table 3). We detected no inconsistency. The beneficial effect of glucocorticosteroids on QoR was clinically plausible. However, all RCTs included in this meta-analysis were at high risk of bias. Serious indirectness was suggested because the included studies did not evaluate the QoR-40 score 3–5 days after surgery. The TSA-adjusted CI was wide, and the cumulative Z-score did not cross the trial sequential monitoring boundary for benefit when our anticipated intervention effect was low. However, using a more optimistic minimal relevant difference of 15 points the analysis demonstrated no imprecision as the trial sequential monitoring boundary for benefit was crossed by the cumulative Z-curve. In addition, we included only three studies; consequently, the “quality of evidence” was graded as “very low”.

**Table 3:** Summary of findings.

Steroids for general anesthesia and surgery				
**Bibliography:**				
**Outcomes**	**No of Participants (studies)**	**Quality of the evidence (GRADE)**	**Anticipated absolute effects**	
	Follow up		**Risk with Control**	**Mean difference with Steroids** (The TSA-adjusted CI)
**Quality of recovery after general anesthesia**^5^	301 (3 studies)	⊕⊝⊝⊝	The mean quality of recovery after general anesthesia in the control groups was	The mean quality of recovery after general anesthesia in the intervention groups was
QoR-40 scale. Scale from: 40 (worst) to 200 (best).		**VERY LOW**^1^^,^^2^^,^^3^^,^^4^	**162 QoR-40 score**	**14.2 higher** (-1.6 to 30 higher)
		due to risk of bias, indirectness, imprecision, publication bias		
**Quality of recovery after general anesthesia (sensitivity analysis**^6^**)**	301 (3 studies)	⊕⊝⊝⊝	The mean quality of recovery after general anesthesia (sensitivity analysis) in the control groups was	The mean quality of recovery after general anesthesia (sensitivity analysis) in the intervention groups was
QoR-40 scale. Scale from: 40 (worst) to 200 (best).		**VERY LOW**^1^^,^^2^^,^^4^	**162 QoR-40 score**	**14.2 higher** (10.4 to 18.1 higher)
		due to risk of bias, indirectness, publication bias		

**TSA**: Trial Sequential Analysis; **CI**: Confidence interval

GRADE Working Group grades of evidence.

**High quality**: Further research is very unlikely to change our confidence in the estimate of effect.

**Moderate quality**: Further research is likely to have an important impact on our confidence in the estimate of effect and may change the estimate.

**Low quality**: Further research is very likely to have an important impact on our confidence in the estimate of effect and is likely to change the estimate.

**Very low quality**: We are very uncertain about the estimate.

^1^ There was no study with low risk of bias in overall domain.

^2^ There was no study which evaluated the QoR-40 score at three days after general anesthesia.

^3^ The TSA-adjusted CI was wide.

^4^ Publication bias could not be assessed because only three trials were included.

^5^ A clinically meaningful anticipated mean difference of the QoR-40 score was set at 5 points.

^6^ A clinically meaningful anticipated mean difference of the QoR-40 score was set at 15 points in the sensitivity analysis.

After adopting a minimal relevant effect of 15 points as a sensitivity analysis, the quality of the evidence was still very low due to high risk of bias; indirectness; and risk of publication bias.

The quality of the evidence on adverse events was also very low due to insufficient reporting; high risk of bias; imprecision, and indirectness problems. Due to the paucity of data, we were not able to conduct TSA.

### Small study effects

We could not perform an asymmetry test for the funnel plot because only three trials were included.

## Discussion

Our meta-analysis indicated that i.v. dexamethasone administration may improve postoperative QoR after general anesthesia. The bias risk assessments and the TSAs revealed that further trials are needed, and the accrued information size reached is only about 15.2% of the estimated RIS based on a minimal relevant difference of 5 points. Based on the GRADE approach, the quality of the assessed evidence was “very low”.

Our study has a number of limitations. First, it is only a meta-analysis focusing on two outcomes, the QoR and adverse events. We need to conduct a systematic review according to the best methodologies [12,14,25,27]. Second, outcomes were only assessed in the short term (one day) after surgery. Third, we only based our evaluation of adverse events on randomized clinical trials. What is needed is also systematic reviews of adverse events based on observational evidence, which picks up rare adverse events as well as late adverse events much better than in randomized clinical trials [21]. Moreover, we did not contact the authors of the trials to get information on unclear or lacking data. Therefore, our current study should be considered as a hypothesis-generating meta-analysis assessing the impacts of potential bias and imprecision on the results of glucocorticosteroids on short-term postoperative symptoms.

The seemingly positive effect of dexamethasone on postoperative QoR is clinically plausible because dexamethasone has anti-emetic effects [39,40] and anti-inflammatory properties that reduce pain [39,41]. The QoR-40 questionnaires assess five dimensions (“physical comfort,” “emotional state,” “physical independence,” “psychological support,” and “pain”). The included randomized clinical trials covered the results of the five dimensions. Two randomized clinical trials [33,34] demonstrated that dexamethasone significantly improved the physical comfort, emotional state, and pain-related dimensions of the QoR-40 score. Another trial [32] established that dexamethasone improved all five dimensions. The dimensions of pain and physical comfort, which included assessments of postoperative nausea and vomiting, were improved in all three studies. Thus, the effects of dexamethasone on pain and postoperative nausea and vomiting played a fundamental role in improving postoperative QoR.

The timing of dexamethasone administration appeared to be homogenous among the three RCTs combined in this meta-analysis. Dexamethasone was administered when the patient was in the pre-operative room [32] or approximately 45 or 60 minutes before the surgical incision [33,34]. It would take 1 to 2 hours for the effects of dexamethasone to appear, because dexamethasone needs to diffuse across the cell membrane and alter gene transcription [42]. Therefore, the administration of glucocorticosteroids at least 45 to 60 minutes before surgery may be a plausible and acceptable strategy for minimizing pain and inflammation [43].

The study by De Oliveira et al.[32] was the only one that investigated dose-dependent effects of dexamethasone on QoR. The authors investigated 0.05 mg · kg^-1^ and 0.1 mg · kg^-1^ dexamethasone dosages. They concluded that the higher dose improved the “physical independence” and “pain” dimensions compared with the lower dose. A recent meta-analysis that evaluated single-dose dexamethasone for postoperative pain also demonstrated that doses exceeding 0.1 mg · kg^-1^ were effective [44]. However, studies investigating the correlation between QoR and dexamethasone dosage are still rare. Therefore, we cannot establish a firm conclusion regarding the optimal dosing of dexamethasone; larger studies are required for this purpose.

Further trials should be conducted to confirm the potential positive effect of dexamethasone on QoR because our finding was affected by high risks of bias as well as high risk of random error in one of the TSA analyses. TSA is able to adjust the CI before the required information size is reached so that type I errors are prevented due to sparse data and multiple testing of the same data [12]. The Z-curve did not cross the TSA monitoring boundary, although it approached the trial sequential boundary for benefit in our most conservative analysis using a minimal relevant difference of 5 points. TSA also revealed that the accrued information size reached only 15.2% of the estimated required information size in this scenario. Choosing a more optimistic minimal relevant difference of 15 points showed that imprecision was not an issue. However, TSA cannot wash away the risks of bias. Furthermore, we were unable to assess small trial effects via a funnel plot because there were only three trials available to be combined. We therefore downgraded the GRADE to “very low”.

In conclusion, our investigation suggests that the perioperative administration of dexamethasone may improve QoR after general anesthesia (GRADE: very low). Further analyses should be performed when the results of additional randomized clinical trials become available. Such trials should include also patients with major abdominal and thoracic surgery. These analyses ought to be conducted as systematic reviews with a public protocol preferably peer reviewed according to the guidelines of Cochrane [14].

## Supporting Information

S1 Supporting Information The search strategy. (DOCX)

S1 Table The PRISMA checklist. (DOC)

S2 Table The reasons for risk of bias assessment. (DOCX)
