# Supplementary material for: Effects of Steroids on Quality of Recovery and Adverse Events after General Anesthesia: Meta-Analysis and Trial Sequential Analysis of Randomized Clinical Trials
Source: PLoS One. 2016 Sep 15;11(9):e0162961. doi: 10.1371/journal.pone.0162961 (PMC5025103; doi:10.1371/journal.pone.0162961)
Supplement: S1 Supporting Information — (DOCX) [file pone.0162961.s001.docx]

S1 Supporting Information. The search strategy

The following PubMed search strategy was established:

(steroid OR steroids OR glucocorticoid OR glucocorticoids OR corticosterone OR corticosteroid OR corticosteroids OR methylprednisolone OR hydrocortisone OR dexamethasone) AND (quality OR enhance OR enhanced) AND recovery) OR QoR OR “quality of recovery”) AND (randomized [tiab] OR randomly [tiab] OR controlled clinical trial [pt] OR randomized controlled trial [pt] OR placebo [tiab] OR drug therapy [sh] OR groups [tiab] OR trial [tiab]) NOT (animals [mh] NOT humans [mh])
