# Supplementary material for: Effects of Steroids on Quality of Recovery and Adverse Events after General Anesthesia: Meta-Analysis and Trial Sequential Analysis of Randomized Clinical Trials
Source: PLoS One. 2016 Sep 15;11(9):e0162961. doi: 10.1371/journal.pone.0162961 (PMC5025103; doi:10.1371/journal.pone.0162961)
Supplement: S2 Table — (DOCX) [file pone.0162961.s003.docx]

S2 Table. The reasons for bias assessment in each trial.

| De Oliveira Jr., 2011[1] |  |  |
| --- | --- | --- |
| Domain | Risk of bias | Reasons |
| Sequence generation | Low | Subjects were randomized using a computer generated table. |
| Allocation concealment | Low | Group assignments were sealed in sequentially numbered opaque envelopes that were opened by a research nurse not involved with the subjects’ care. |
| Patients blinded | Low | This was a well-designed double-blind placebo controlled trial. |
| Health care providers blinded | Low | This was a well-designed double-blind placebo controlled trial. |
| Data collectors blinded | Low | This was a well-designed double-blind placebo controlled trial. |
| Outcome assessors blinded | Low | This was a well-designed double-blind placebo controlled trial. |
| Incomplete outcome data | Low | Number of missing data balanced across groups. |
| Selective reporting | Unclear | Severe adverse events were not evaluated. |
| Other bias | Low | We found no other bias. |
| Overall risk | High | There was "Unclear" domain. |

| Murphy, 2011a [2] |  |  |
| --- | --- | --- |
| Domain | Risk of bias | Reason |
| Sequence generation | Low | Computer generated randomization code was used. |
| Allocation concealment | Low | Study medications were prepared by the operating room pharmacy in 3-ml syringes labeled with the patient’s name. |
| Patients blinded | Low | This study was a well-designed double-blind, placebo-controlled investigation. |
| Health care providers blinded | Low | This study was a well-designed double-blind, placebo-controlled investigation. |
| Data collectors blinded | Low | This study was a well-designed double-blind, placebo-controlled investigation. |
| Outcome assessors blinded | Low | This study was a well-designed double-blind, placebo-controlled investigation. |
| Incomplete outcome data | Unclear | Missing data exceeded 20%, although number of missing data balanced across groups. |
| Selective reporting | Unclear | It is unclear whether they surveyed postoperative infection in the word. |
| Other bias | Low | We found no other bias. |
| Overall risk | High | There were "Unclear" domains. |

| Murphy, 2011b [3] |  |  |
| --- | --- | --- |
| Domain | Risk of bias | Reasons |
| Sequence generation | Low | Computer generated randomization code was used. |
| Allocation concealment | Low | Study medications were prepared by the operating room pharmacy in 3-ml syringes labeled with the patient’s name. |
| Patients blinded | Low | This study was a well-designed double-blind, placebo-controlled investigation. |
| Health care providers blinded | Low | This study was a well-designed double-blind, placebo-controlled investigation. |
| Data collectors blinded | Low | This study was a well-designed double-blind, placebo-controlled investigation. |
| Outcome assessors blinded | Low | This study was a well-designed double-blind, placebo-controlled investigation. |
| Incomplete outcome data | Unclear | Number of missing data imbalanced. |
| Selective reporting | Low | All of the study’s prespecified outcomes and all expected outcomes of interest including infection or hyperglycemia were reported. |
| Other bias | Unclear | Number of participants assigned to each group imbalanced. |
| Overall risk | High | There were "Unclear" domains. |

| Pauls, 2015 [4] |  |  |
| --- | --- | --- |
| Domain | Risk of bias | Reasons |
| Sequence generation | Low | Computer generated randomization table was used. |
| Allocation concealment | Low | The hospital pharmacy was provided a confidential table to allocate each patient. |
| Patients blinded | Low | The patient, physicians, anesthesia personnel, nursing, data collection staff, and statistician were all blinded. |
| Health care providers blinded | Low | The patient, physicians, anesthesia personnel, nursing, data collection staff, and statistician were all blinded. |
| Data collectors blinded | Low | The patient, physicians, anesthesia personnel, nursing, data collection staff, and statistician were all blinded. |
| Outcome assessors blinded | Low | The patient, physicians, anesthesia personnel, nursing, data collection staff, and statistician were all blinded. |
| Incomplete outcome data | High | Number of the dexamethasone group was 25% less than the control group probably because number of missing data imbalanced across groups. |
| Selective reporting | Unclear | Severe adverse events were not evaluated. |
| Other bias | High | The QoR-40 score in pain domain improved after surgery even in the control group. |
| Overall risk | High | There were "High" domains. |

**References**

1. De Oliveira GS Jr, Ahmad S, Fitzgerald PC, Marcus RJ, Altman CS, Panjwani AS, et al. Dose ranging study on the effect of preoperative dexamethasone on postoperative quality of recovery and opioid consumption after ambulatory gynaecological surgery. Br J Anaesth. 2011;107: 362-371. doi:10.1093/bja/aer156

2. Murphy GS, Szokol JW, Greenberg SB, Avram MJ, Vender JS, Nisman M, et al. Preoperative dexamethasone enhances quality of recovery after laparoscopic cholecystectomy: effect on in-hospital and postdischarge recovery outcomes. Anesthesiology. 2011;114: 882-890. doi:10.1097/ALN.0b013e3181ec642e

3. Murphy GS, Sherwani SS, Szokol JW, Avram MJ, Greenberg SB, Patel KM, et al. Small-dose dexamethasone improves quality of recovery scores after elective cardiac surgery: a randomized, double-blind, placebo-controlled study. J Cardiothorac Vasc Anesth. 2011;25: 950-960. doi:10.1053/j.jvca.2011.03.002

4. Pauls RN, Crisp CC, Oakley SH, Westermann LB, Mazloomdoost D, Kleeman SD, et al. Effects of dexamethasone on quality of recovery following vaginal surgery: a randomized trial. Am J Obstet Gynecol. 2015;213: 718.e1–7. doi:10.1016/j.ajog.2015.05.061
